# Aguamiel concentrate from *Agave salmiana* and its extracted saponins attenuated obesity and hepatic steatosis and increased *Akkermansia muciniphila* in C57BL6 mice

**DOI:** 10.1038/srep34242

**Published:** 2016-09-28

**Authors:** Ana María Leal-Díaz, Lilia G. Noriega, Ivan Torre-Villalvazo, Nimbe Torres, Gabriela Alemán-Escondrillas, Patricia López-Romero, Mónica Sánchez-Tapia, Miriam Aguilar-López, Janette Furuzawa-Carballeda, Laura A. Velázquez-Villegas, Azalia Avila-Nava, Guillermo Ordáz, Janet A. Gutiérrez-Uribe, Sergio O. Serna-Saldivar, Armando R. Tovar

**Affiliations:** 1 Tecnologico de Monterrey, Centro de Biotecnología FEMSA, Escuela de Ingeniería y Ciencias, 64849, Monterrey, NL, Mexico; 2 Departamento de Fisiología de la Nutrición, Instituto Nacional de Ciencias Médicas y Nutrición Salvador Zubirán, 14080, México D.F., Mexico; 3 Departamento de Inmunología y Reumatología, Instituto Nacional de Ciencias Médicas y Nutrición Salvador Zubirán, 14080, México D.F., Mexico

## Abstract

Obesity and its comorbidities are a severe public health problem worldwide. The use of bioactive compounds found in some foods has been demonstrated to ameliorate the metabolic abnormalities associated with obesity. The purpose of this study was to assess whether the bioactive compounds present in aguamiel concentrate (AC) from *Agave salmiana* could attenuate glucose intolerance and hepatic steatosis in mice fed a high fat (HF) diet. HPLC-ELSD analysis showed that AC contained several saponins. The consumption of an AC extract rich in saponins reduced weight gain and fat mass and lowered serum glucose, insulin and LDL-cholesterol levels in mice fed a HF diet. Additionally, mice fed the saponin extract exhibited a reduced HOMA index and hepatic lipid levels and increased expression of genes involved in fatty acid oxidation. Saponins increased white adipose tissue browning, AMPK phosphorylation, fatty acid oxidation, and mitochondrial activity in skeletal muscle and energy expenditure in mice fed the HF diet. These metabolic changes were accompanied by an increase in the abundance of *Akkermansia muciniphila* in the gut microbiota. Therefore, *Agave salmiana* saponins can be an alternative to attenuate the metabolic changes that accompany obesity.

Since 1980, obesity prevalence has doubled worldwide. In 2014, overweight individuals represented 39% of the adult population, and 13% of these individuals were obese^1^. Specific metabolic abnormalities develop in obese individuals, such as pro-inflammatory states, dyslipidemia, high blood pressure, insulin resistance, glucose intolerance and non-alcoholic fatty liver disease (NAFLD); these abnormalities are accompanied by gut microbiota dysbiosis^2^,^3^. Insulin resistance develops during obesity due to alterations in insulin signaling and increases in the systemic inflammatory response^4^. These alterations occur in part through a lipotoxic effect due to the accumulation of lipids in non-adipose tissue organs, particularly the liver and skeletal muscle^5^.

Recently, it has been established that dietary intervention must be included to prevent or ameliorate the biochemical abnormalities associated with obesity^6^. With this aim, extensive research on functional foods has recently been performed. These foods provide a health benefit in addition to their nutritional value^7^. Most properties of functional foods are associated with the presence of specific bioactive compounds that regulate precise pathways to exert their beneficial effect^8^.

In Mexico, several functional foods have been used to obtain health benefits. For example, agave (*Agave* spp.) has been used since Pre-Columbian times as a food and beverage source^9^,^10^. Currently, agave possesses economic relevance in the tequila, mezcal and pulque industries^10^. Aguamiel is the edible sweet sap obtained from specific mature agave species such as *Agave salmiana*^11^. It may be consumed fresh, fermented or concentrated by heat into a syrup or aguamiel concentrate (AC)^11^. In Mexico, diabetic people consume AC to improve their diabetic condition. The testimonials reported by consumers suggest an improvement in glycemic control. Nevertheless, AC has not been studied to assess its biological effects.

Scarce knowledge is available concerning the bioactive compounds present in aguamiel. Ortiz-Basurto *et al.* (2012) evaluated fresh aguamiel and reported that it contained 11.5% dry matter that was mainly composed of sugars; a total of 10% of these sugars were fructans. Aguamiel also contained 3% protein, 3% minerals, and 0.3% free amino acids^12^. Recently, our research group demonstrated that AC also contained steroidal saponins^11^,^13^. These molecules are a diverse group of biologically active glycosides that contribute to the plant’s defense and are widely distributed in the plant kingdom^13^. Different saponins have been used to treat obesity. Some of the most recognized are the saponins from *Panax ginseng*, *Panax japonicas*, and *Platycodi radix*, which have been validated in different models to prevent or decrease obesity^8^,^14^,^15^,^16^.

Dioscin is a steroidal saponin whose oral consumption has been demonstrated to prevent diet-induced obesity and non-alcoholic fatty liver disease by increasing the energy expenditure^17^. However, scant knowledge is available concerning the potential mechanisms of action of many saponins and less is known about those present in aguamiel. Thus, the aim of the present study was to assess the effect of the consumption of AC from *Agave salmiana* and an AC extract rich in saponins in mice fed a high fat diet (HF) to evaluate their effects on carbohydrate/lipid metabolism and the gut microbiota composition. Here, we demonstrate that an AC extract rich in saponins improves glucose tolerance and serum and hepatic lipid levels, induces WAT browning and mitochondrial activity in skeletal muscle by increasing energy expenditure, and increases *Akkermansia muciniphila* in the gut microbiota of mice fed the HF diet.

## Results and Discussion

### Aguamiel concentrate’s available carbohydrate composition

**Figure 1:**
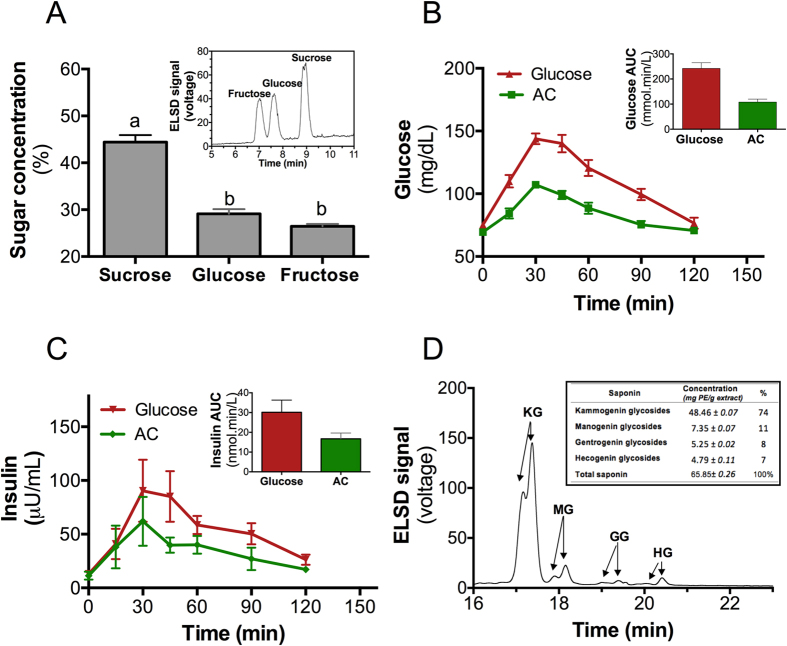
Aguamiel concentrate had low glycemic and insulinemic indices in humans and contains steroidal saponins. (**A**) AC sugar quantification and its corresponding HPLC-ELSD chromatogram (inset). (**B**) Blood glucose (**C**) and serum insulin levels during the fasting state and 15, 30, 45, 60, 90, 120 min after the consumption of 50 g of oral glucose or 50 g of available carbohydrates from AC and their corresponding incremental areas under the curve (AUC) (insets). (**D**) HPLC-ELSD steroidal saponin profile and quantification (inset).

To obtain AC, the plant is not harvested. Instead, aguamiel is collected daily from the core of *Agave salmiana* and concentrated using heat. The AC contained a high concentration of available carbohydrates (46.3%). HPLC-ELSD analysis revealed that the AC was primarily composed of sucrose (44.4%), glucose (29.1%) and fructose (26.5%) (Fig. 1A); the minor components are described in Supplemental Table S1. The AC composition differed from the high fructose agave syrup from *Agave tequilana*^10^,^18^.

### Aguamiel concentrate has low glycemic and insulinemic indices

To investigate whether the available carbohydrates in AC could modify blood glucose, we studied its glycemic and insulinemic indices. Interestingly, we observed that AC produced a lower increase in blood glucose and serum insulin compared with the response to the reference (50 g of glucose) despite the elevated sucrose and glucose contents in the 50 g of available carbohydrates in AC (Fig. 1A–C). Measurement of the area under the curve (AUC) proved that the AC had low glycemic and insulinemic indices (47.6 and 53, respectively) as was previously reported for high-fructose agave syrup^19^. Thus, we hypothesized that compounds other than sugars were responsible for its low GI and performed a composition analysis of the AC.

### Bioactive saponins in aguamiel concentrate

The presence of steroidal saponins in the AC was analyzed by HPLC-ELSD. We detected saponins derived from kammogenin, manogenin, gentrogenin and hecogenin, which represented 74, 11, 8 and 7% of the total saponins, respectively (Fig. 1D). Saponins present in other natural products are capable of decreasing the acute glycemic response^20^,^21^. Therefore, we extracted the saponins with a mixture of *n*-butanol/H_2_O to assess the biological activity of these compounds on carbohydrate/lipid metabolism in mice fed a high-fat (HF) diet. This extract was provided in the HF diet at a low dose (HFL) by adding 2.8 g/kg diet or at a high dose (HFH) by adding 28 g/kg diet (HFH).

### Aguamiel concentrate and its extracted saponins prevent body weight gain in a high fat diet

**Figure 2:**
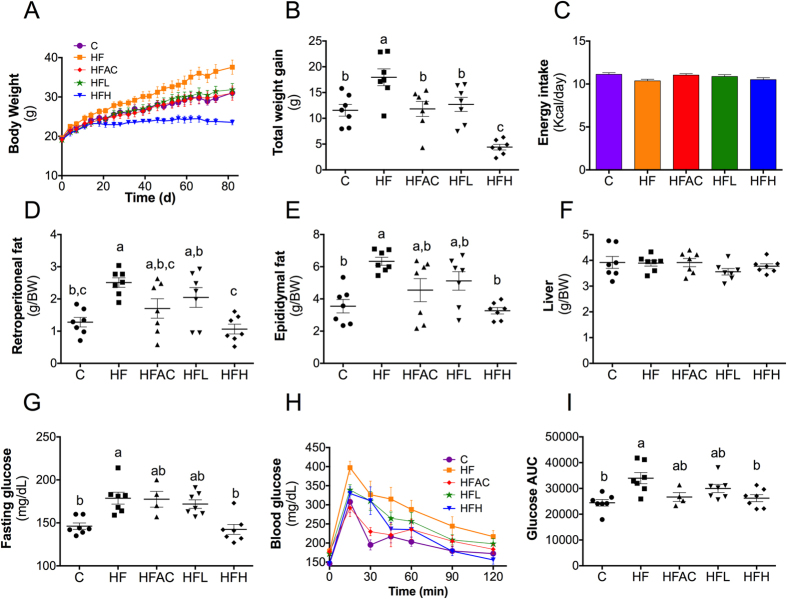
Aguamiel concentrate and its saponins prevented obesity and glucose intolerance in C57BL6 mice. (**A**) Growth curve, (**B**) total weight gain, and (**C**) daily average energy intake after 12 weeks of diet-induced obesity. Animals were housed in groups (3–4 mice per cage), food intake was recorded five times per week and animal weight twice per week. (**D**) Retroperitoneal fat tissue weight, (**E**) epididymal fat tissue weight and (**F**) liver tissue weight normalized to total body weight after 12 weeks of diet-induced obesity. Effect of AC or its saponins on (**G**) the fasting glucose level, (**H**) OGTT and (**I**) glucose AUC after 10 weeks of diet-induced obesity. Data are presented as the mean ± SEM (n = 4–7). Statistical analysis was performed with one-way ANOVA followed by Tukey’s *post hoc* test. Treatments with different letter where a > b > c are significantly different at *P* < 0.05. C: control; HF: High-fat; HFAC: High-fat with aguamiel concentrate; HFL: High-fat with low saponin dose; HFH: High-fat with high saponin dose.

As expected, mice fed the HF diet gained more weight (55%) compared to the control (C) group (Fig. 2A,B). In contrast, mice fed the HF diet supplemented with AC (HFAC) and HFL gained weight at a level comparable to their C counterparts. Moreover, mice fed HFH gained less weight compared to the C group, denoting a dose-dependent effect. The weight difference was not an effect of a decrease in the energy intake because it was similar in all groups (Fig. 2C). Additionally, the weight difference was not associated with an inhibitory effect of saponins on pancreatic lipase as previously suggested^15^,^22^ because there was no difference in the fecal fat content (Supplemental Fig. S1). Consistent with the weight gain, the visceral adipose tissue weight (retroperitoneal and epididymal) was greater in animals fed the HF diet compared to the animals fed the C diet (Fig. 2D,E). Interestingly, the adiposity of the mice fed HFAC, HFL and HFH was similar to the C group despite the HF diet. This effect was more pronounced in the retroperitoneal adipose tissue where it represented up to 17.9% of the body weight in the HF group compared to 4.4% of the body weight in the HFH group. Finally, the liver weight was not affected by the experimental diets (Fig. 2F).

### Aguamiel concentrate and its extracted saponins prevent an increase in LDL-cholesterol and HOMA

Plasma samples were analyzed to evaluate the effect of AC or its extracted saponins on the biochemical parameters of the treated mice; the results are shown in Table 1. After 12 weeks on the experimental diets, within the serum lipid profile, the total cholesterol, HDL-cholesterol, triacylglyceride (TAG) concentrations as well as glucose concentration were not significantly (*P* < 0.05) different among the treatment groups. Conversely, the HF group had greater LDL-cholesterol (LDL-C) and insulin concentrations compared to the C group. When AC or its extracted saponins were added to the HF diet, the increase in the plasma LDL-C and insulin concentrations was prevented. Interestingly, HOMA-IR was lower in the HFH group, suggesting that saponins could have an effect on glucose tolerance.

### Aguamiel concentrate and its extracted saponins improve glucose tolerance

To evaluate whether the AC and its extracted saponins had an effect on glucose clearance, an oral glucose tolerance test (OGTT) was performed during the 10^th^ week of the experiment. As expected, animals fed the HF diet exhibited a significant (*P* < 0.05) increase in their blood glucose compared to the mice fed the C diet both at the fasting state and during the OGTT, indicating that the HF group had decreased glucose tolerance (Fig. 2G–I). Interestingly, mice fed the HFAC and HFH diets exhibited significantly (*P* < 0.05) increased glucose tolerance as observed by the lower area under the curve (AUC) compared to the mice fed the HF diet (Fig. 2I). To assess whether consumption of the saponin extract could also reduce body weight and glucose intolerance in obese mice, we fed mice a HFD for 16 weeks to establish obesity and hyperglycemia. Subsequently, the mice received HFH for another 8 weeks. As shown in Supplemental Fig. S2, during the first 16 weeks on the HF diet the mice became obese and hyperglycemic. Interestingly, this preliminary study shows that the mice lost 28% of their body weight 8 weeks after switching to the diet with HFH. Additionally, the glucose tolerance was remarkably increased and the AUC was decreased by 47.5%. Further research is needed using different doses in order to understand the mechanism of action.

### Aguamiel concentrate or its extracted saponins prevents hepatic steatosis

**Figure 3:**
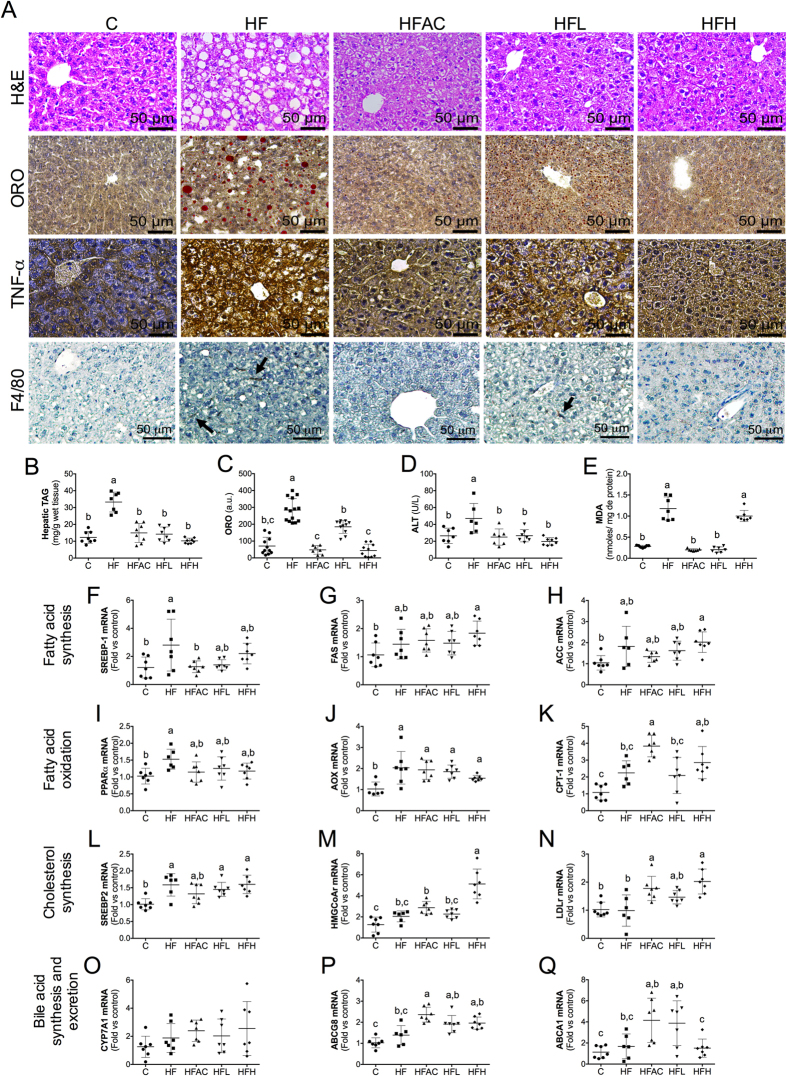
Aguamiel concentrate and its saponins prevented hepatic steatosis and regulated the expression of genes related to lipid metabolism in C57BL6 mice. (**A**) Histopathological examination with H&E, Oil Red O (ORO) staining, and TNF-α and F4/80 immunohistochemical staining (400x magnification). (**B**) Total hepatic triacylglycerides (TAG) quantification, (**C**) ORO staining quantification, (**D**) plasma alanine-aminotransferase (ALT) levels, and (**E**) malondialdehyde (MDA) content in liver. Effect of AC and its extracted saponins on hepatic gene expression of (**F**) SREBP1, (**G**) FAS, (**H**) ACC, (**I**) PPARα, (**J**) AOX, (**K**) CPT-1, (**L**) SREBP2, (**M**) HMGCR, (**N**) LDLr, (**O**) CYP7A1, (**P**) ABCG8, and (**Q**) ABCA1. The cDNA was analyzed per mouse. Statistical analysis for (**B**–**Q**) was performed with one-way ANOVA followed by a Tukey’s *post hoc* test. Mean values with different letters where a > b > c are significantly different (at least *P* < 0.05). Data are expressed as the mean ± SEM. C: control; HF: High-fat; HFAC: High-fat with aguamiel concentrate; HFL: High-fat with low saponin dose; HFH: High-fat with high saponin dose.

Glucose intolerance is normally accompanied by hepatic steatosis^23^. To evaluate whether AC or its extracted saponins had an impact on hepatic steatosis, we evaluated the liver morphology. Liver histological analysis using hematoxylin and eosin (H&E) as well as Oil Red O (ORO) staining clearly shows that mice fed the HF diet had a greater hepatic fat accumulation in the form of macro- and microvesicles compared to the C group (Fig. 3A). Notably, hepatic lipid accumulation decreased when AC was added to the diet; this effect was similar in a dose-dependent manner with the saponin extract. The hepatic steatosis observed in the histological analysis was confirmed by the quantification of hepatic TAG (Fig. 3B), and ORO image analysis (Fig. 3C). The livers of mice fed the HF diet showed a significantly (*P* < 0.005) higher TAG accumulation compared to mice fed the C diet. As expected, consumption of AC or its extracted saponins prevented hepatic TAG accumulation. To evaluate hepatic inflammation, we assessed the expression of tumor necrosis factor-α (TNF-α) by immunohistochemistry. Mice fed the HF diet had increased levels of TNF-α compared to mice fed the C diet (Fig. 3A). Moreover, the addition of AC or its extracted saponins reduced hepatic TNF-α compared to the HF group in a dose-dependent manner. To further evaluate if macrophage infiltration was also affected, we then evaluated F4/80 by immunohistochemistry (Fig. 3A). Results showed that mice fed the HF diet had a greater macrophage infiltration compared to mice fed the control diet. This effect was prevented in HFAC and HFH groups. Consumption of a HF diet also increased significantly (*P* < 0.05) the plasma alanine aminotransferase (ALT) level compared to the C group (Fig. 3D). The addition of AC or the saponin extract did not increase the ALT content, which ruled out a hepatotoxic effect. The liver TAG and ALT levels showed a positive correlation (r^2^ = 0.657) (Supplemental Fig. S3). To assess oxidative stress, we measured the malondialdehyde (MDA) content in the liver. The results showed that MDA was increased in the HF group compared to the C group; interestingly, this effect was reversed in the HFAC and HFL groups (Fig. 3E). However, in the HFH group this effect was not reverted. There is evidence that at higher doses or under certain conditions antioxidant-type functional food ingredients may exert pro-oxidant activities^24^. Thus, it is possible that the use of the high dose of the saponin extract could have a pro-oxidant activity.

### Aguamiel concentrate or its extracted saponins regulates the expression of hepatic genes involved in lipid metabolism

To evaluate whether the decrease in hepatic TAG was associated with changes in the expression of genes involved in hepatic lipid metabolism, we evaluated their relative expression levels. As expected, mice fed the HF diet exhibited up-regulated expression of lipogenic genes, such as the transcription factor sterol regulatory element-binding protein–1c (SREBP-1c), fatty acid synthase (FAS) and acetyl-CoA carboxylase (ACC) (Fig. 3F–H). Interestingly, the addition of AC or the saponin extract to the HF diet decreased the expression of SREBP1c to a level similar to the C group. Next, we studied the expression of genes involved in fatty acid oxidation, particularly the transcription factor peroxisome proliferator-activated receptor-α (PPAR-α) and two of its target genes [carnitine palmitoyl transferase-1 (CPT-1) and acyl CoA oxidase (AOX)]. In Fig. 3I–K, we showed that CPT-1 expression levels was similar between the HF group and the C group. However, the HFAC group exhibited significantly (*P* < 0.05) increased expression of CPT-1, which was 4-fold higher than the C group. These results suggest that the AC can increase hepatic fatty acid oxidation and therefore reduce hepatic lipids. With respect to cholesterol metabolism, we evaluated the expression of transcription factor sterol regulatory element-binding protein–2 (SREBP-2), the enzyme HMG-CoA reductase and the LDL receptor (LDL-R). There was a significant up-regulation of SREBP-2 and LDL-R expression in mice fed the HF diet compared to the C group. When the saponin extract was added to the HF diet, the SREBP-2, HMG-CoA reductase and LDL-R expression levels were up-regulated compared to the HF group (Fig. 3L–N). These results suggest that even though cholesterol production was not downregulated, LDL-C uptake by the hepatocyte was increased when saponins were added into the diet, which may explain the decrease in plasma LDL-C. Furthermore, the expression of the enzyme cholesterol 7 α-hydroxylase (CYP7A1) which is involved in the synthesis of bile acids, was also up-regulated in mice fed the HF diets and was further up-regulated in mice fed HFH (Fig. 3O). Lastly, the transporters involved in bile acid and cholesterol transport, ATP-binding cassette transporters (ABCG8 and ABCA1) were up-regulated in the livers of mice fed HFAC, HFL and HFH compared to mice fed the control and HF diets, indicating greater cholesterol excretion (Fig. 3P,Q). These results indicate that the AC extract rich in saponins stimulates fat oxidation and cholesterol excretion, contributing in part to the amelioration of hepatic steatosis. Other saponins (i.e., dioscin) have the capacity to reduce or prevent hepatic steatosis through a direct interaction with the nuclear receptor PPARα and thereby increase fatty acid oxidation in the liver and skeletal muscle^17^.

### Aguamiel concentrate and its extracted saponins prevent white adipose tissue (WAT) hypertrophy

**Figure 4:**
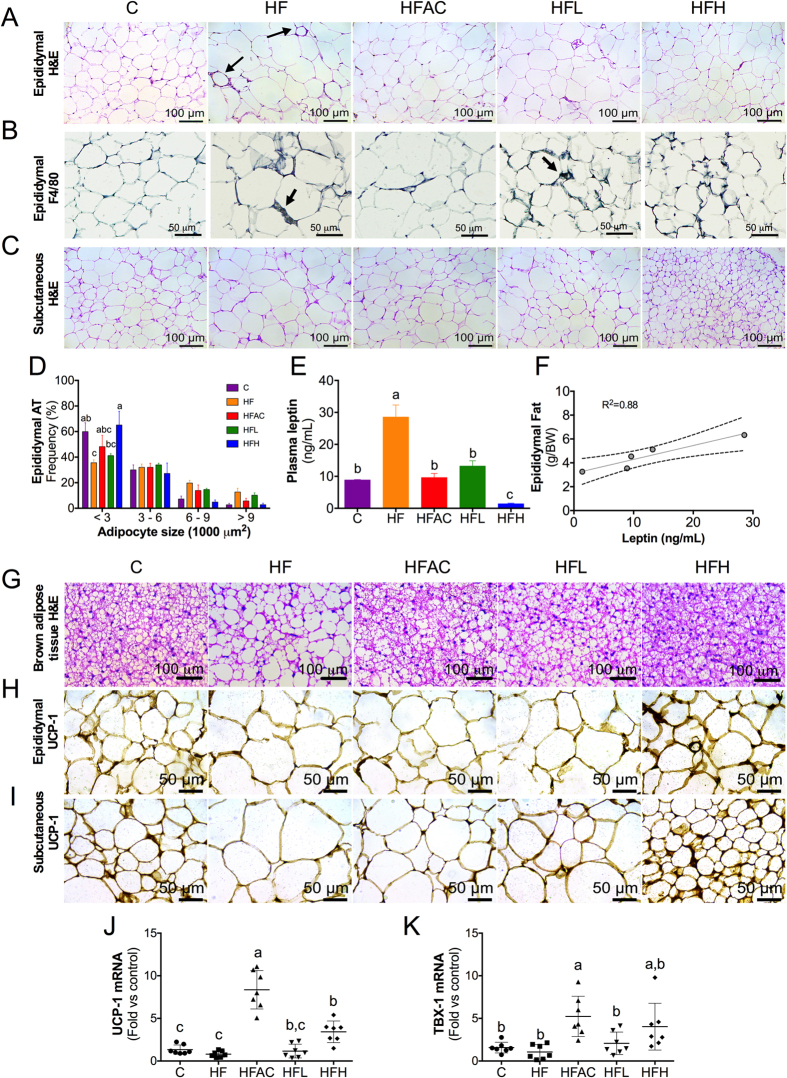
Aguamiel concentrate and its saponins prevented adipose tissue hypertrophy, hyperleptinemia, and increased UCP-1 expression. (**A**) H&E staining of epididymal adipose tissue (200x magnification). (**B**) Immunohistochemical determination of F4/80-positive cells in epididymal adipose tissue (400x magnification). (**C**) H&E staining of subcutaneous adipose tissue (200x magnification). (**D**) Epididymal adipocyte size distribution. (**E**) Plasma leptin concentration and (**F**) correlation between epididymal fat and plasma leptin. (**G**) Brown subcutaneous adipose tissue H&E staining (200x magnification). (**H**) Epididymal and (**I**) subcutaneous UCP-1 immunohistochemical staining (400x magnification). Subcutaneous adipose tissue gene expression of (**J**) UCP1, and (K) TBX1. The statistical analysis was performed with one-way ANOVA followed by the Bonferroni *post hoc* test. Mean values with different letters where a > b > c at the same adipocyte size are significantly different (at least *P* < 0.05). C: control; HF: High-fat; HFAC: High-fat with aguamiel concentrate; HFL: High-fat with low saponin dose; HFH: High-fat with high saponin dose.

Because AC or its extracted saponins reduced weight gain despite the consumption of a HF diet, we evaluated the effect of AC or its saponin extract on adipocyte hypertrophy. Mice that consumed the HF diet presented enlarged adipocytes in all adipose compartments compared to mice fed the C diet (Fig. 4A,C). Moreover, crown-like structures, with the presence of macrophages confirmed by F4/80 immunohistochemistry on the epididymal fat, were mostly observed in mice fed the HF diet, followed by mice fed the HFL diet (Fig. 4B). The crown-like structures are formed by the recruitment of macrophages into white adipose tissue in response to adipocyte death by necrosis^25^. Interestingly, the addition of AC or its saponin extract to the HF diet prevented adipocyte hypertrophy and no crown-like structures were found across the different adipose tissue depots. Automatized visceral adipocyte area analysis confirmed a decreased proportion of small adipocytes (area <3,000 μm^2^) in mice fed the HF diet (35.6%) compared to the C group (60.0%) (Fig. 4D). Furthermore, the hypertrophic adipocytes (area >9,000 μm^2^) increased from 2.8% in the C group to 12.7% in the HF group. The addition of AC or the high dose of the saponin extract prevented adipocyte hypertrophy and increased the proportion of small adipocytes regardless of the high fat content in the diet, particularly in the HFH group. Information on retroperitoneal adipose tissue is available in Supplemental Fig. S4.

Leptin is secreted from adipocytes in proportion to the adipose tissue mass^26^. This process was observed in our study, with circulating leptin increased 3-fold in mice fed the HF diet compared to mice fed the C diet (Fig. 4E). Mice fed HFAC, HFL and HFH had reduced circulating leptin levels that corresponded to the adipose tissue mass and adipocyte size (Fig. 4F).

### Aguamiel concentrate and its extracted saponins reduce brown adipose tissue (BAT) lipid droplet size and induce WAT browning

As observed in Fig. 4G, the BAT from mice fed the C diet was composed of multilocular adipocytes with numerous and small lipid vacuoles, whereas the BAT from mice fed the HF diet contained several adipocytes with one single large lipid vacuole. The BAT from mice fed the HFAC showed fewer unilocular and higher multilocular adipocytes content than the HF group. The addition of the saponin extract had a remarkable dose-dependent effect on BAT morphology. Brown adipocytes from mice fed the HFL diet had smaller lipid droplets than those from mice fed the HF diet; brown adipocytes from mice fed HFH had almost imperceptible lipid droplets, indicating robust lipid oxidation^27^.

Body fat gain on a high fat diet can be reduced by an increase in white browning, thereby augmenting the mitochondrial uncoupling protein-1 (UCP-1) content^28^. UCP-1 in beige adipocytes impairs electron transport chain-mediated ATP synthesis and dissipates metabolic energy as heat^29^. To evaluate WAT browning, UCP-1 expression was assessed using immunohistochemical analysis. Interestingly, UCP-1 expression was reduced in epididymal and subcutaneous adipose tissue from mice fed the HF diet compared to the C group (Fig. 4H,I). However, the addition of AC or its saponin extract to the HF diet increased UCP-1 abundance in a dose-dependent manner. The increase of browning by the AC and the high dose of the saponin extract in the subcutaneous adipose tissue was confirmed by the increase of UCP1 and TBX1 mRNA abundance (Fig. 4J,K), markers of the browning process^29^. These results indicate that the reduction in body fat in mice fed the HF diet supplemented with AC or the saponin extract was in part associated with an increase in white adipose tissue browning.

### Aguamiel concentrate and its saponin extract increase energy expenditure

**Figure 5:**
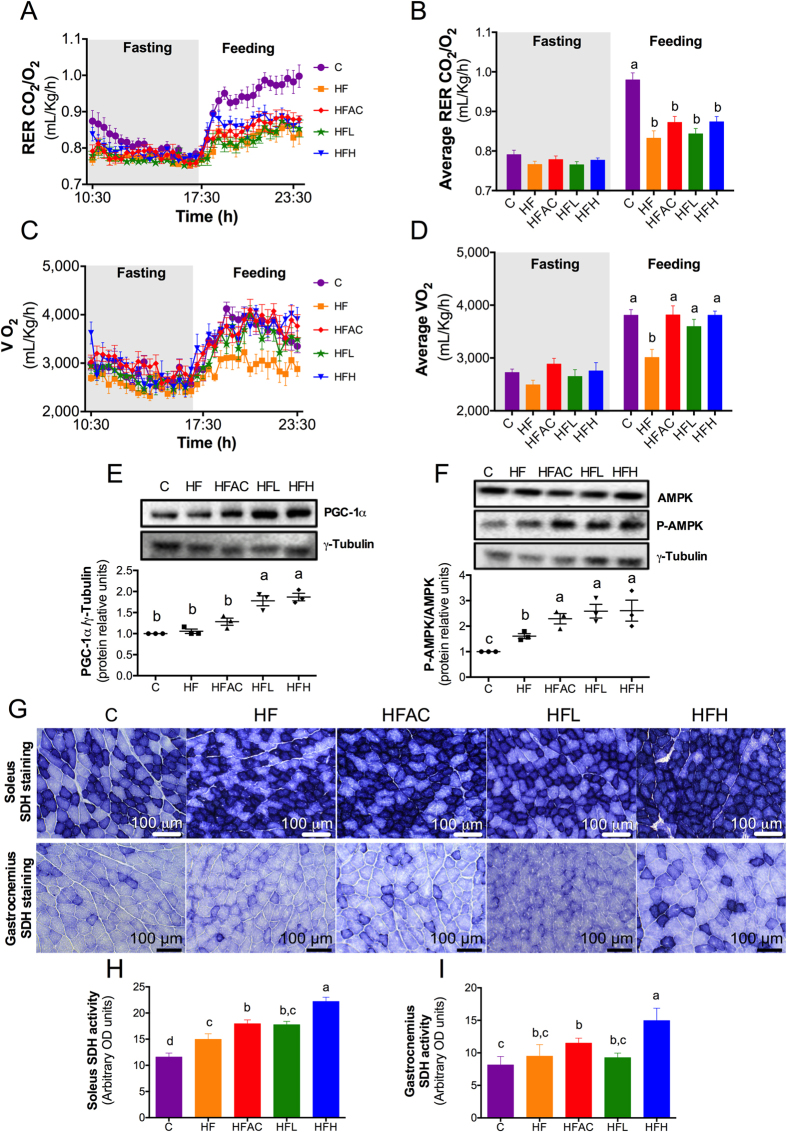
Aguamiel concentrate and its saponins increased energy expenditure and muscle oxidative capacity in C57BL6 mice. (**A**,**B**) Respiratory exchange ratio (RER) and (**C**,**D**) oxygen consumption during the fasting and feeding stages. Relative protein expression for (**E**) PGC-1α and (**F**) the phosphorylated AMPK ratio (P-AMPK/AMPK). Skeletal muscle tissue histochemically stained for succinate dehydrogenase (SDH) activity in (**G**) the soleus and gastrocnemius. Quantification of SDH staining in (**H**) soleus and (**I**) gastrocnemius. Data are expressed as the mean ± SEM. Statistical analysis was performed with one-way ANOVA followed by Tukey’s *post hoc* test. Mean values with different letters where a > b > c are significantly different at *P* < 0.05. C: control; HF: High-fat; HFAC: High-fat with aguamiel concentrate; HFL: High-fat with low saponin dose; HFH: High-fat with high saponin dose.

To evaluate whether changes in BAT morphology and WAT browning were associated with changes in energy expenditure, we performed an indirect calorimetry during the fasting and feeding states after 11 weeks on the experimental diet. First, we assessed whether consumption of AC or the saponin extract modified the type of metabolic substrate. Based on the respiratory exchange ratio (RER) measurement, there were no significant (*P* < 0.05) differences among groups during fasting (RER 0.76–0.79), indicating that the animals were obtaining their energy primarily from fatty acids (Fig. 5A,B). After the animals were fed, the C group RER increased to 0.97, whereas the HF group RER increased to only 0.83, indicating that mice fed the HF diet developed metabolic inflexibility compared to the C group^30^. AC or the saponin extract did not modify the RER in mice fed the HF diet. However, mice fed the HF diet consumed 21% less O_2_ compared to the C group when we evaluated the energy expenditure via O_2_ consumption during the fed state (Fig. 5C,D). Interestingly, when AC or its saponin extract were added to the HF diet the O_2_ consumption was similar to the C group. Thus, the HFAC and HFH groups increased the O_2_ consumption by 27% and the HFL group increased it by 19% compared to the HF group. These results demonstrated that AC and its saponin extract increased the energy expenditure despite the HF content of the diet, as was observed with other saponins such as dioscin^17^ and the gingenoside Rb1^31^.

### Aguamiel concentrate and its saponin extract increase mitochondrial activity in skeletal muscle

To evaluate whether the increase in energy expenditure in mice fed AC or the saponin extract was also associated with greater mitochondrial activity in skeletal muscle, we measured the expression of the peroxisome proliferator-activated receptor gamma coactivator 1-α (PGC1-α), which is a nuclear receptor-coactivator involved in mitochondrial biogenesis and function^28^. Mice fed the C and the HF diet had similar PGC1-α protein expression levels (Fig. 5E). Interestingly, mice fed the saponin extract showed an increase in the PGC1-α protein content.

To enhance PGC1-α activity, it is necessary to activate the enzyme 5′ adenosine monophosphate-activated protein kinase (AMPK) by phosphorylation^32^. In this study, AC and especially the saponin extract increased the activation of AMPK by phosphorylation of the Thr172 residue, indicating an increase in fatty acid oxidation (Fig. 5F). Indeed, we observed an increase in the mitochondrial activity in the soleus and gastrocnemius skeletal muscles measured histochemically through succinate dehydrogenase (SDH) activity, particularly in mice fed HFH (Fig. 5G–I). These results suggest that the AC saponin extract is able to stimulate mitochondrial activity in skeletal muscle.

### Aguamiel concentrate or the saponin extract differentially modulate the intestinal microbiota composition

Some of the beneficial effects of the aguamiel concentrate or the saponin extract could be attributed to modifications of the gut microbiota due to the prebiotic potential of agave and possible direct effects of saponins^33^. Thus, we assessed the gut microbiota by sequencing the 16S rRNA gene. Illumina MiSeq sequencing of the samples resulted in >50,000 reads. Bacteroidetes, Firmicutes and Proteobacteria represented ~94% of the sequences of all groups at the phylum level. Alpha diversity measures such as the Chao 1 estimator suggested that the observed OTUs were higher in the HFH group, whereas the Shannon index was higher in the C group. These results were confirmed using rarefaction curve analysis that indicated higher species richness in the HFH group and higher diversity in the C group.

**Figure 6:**
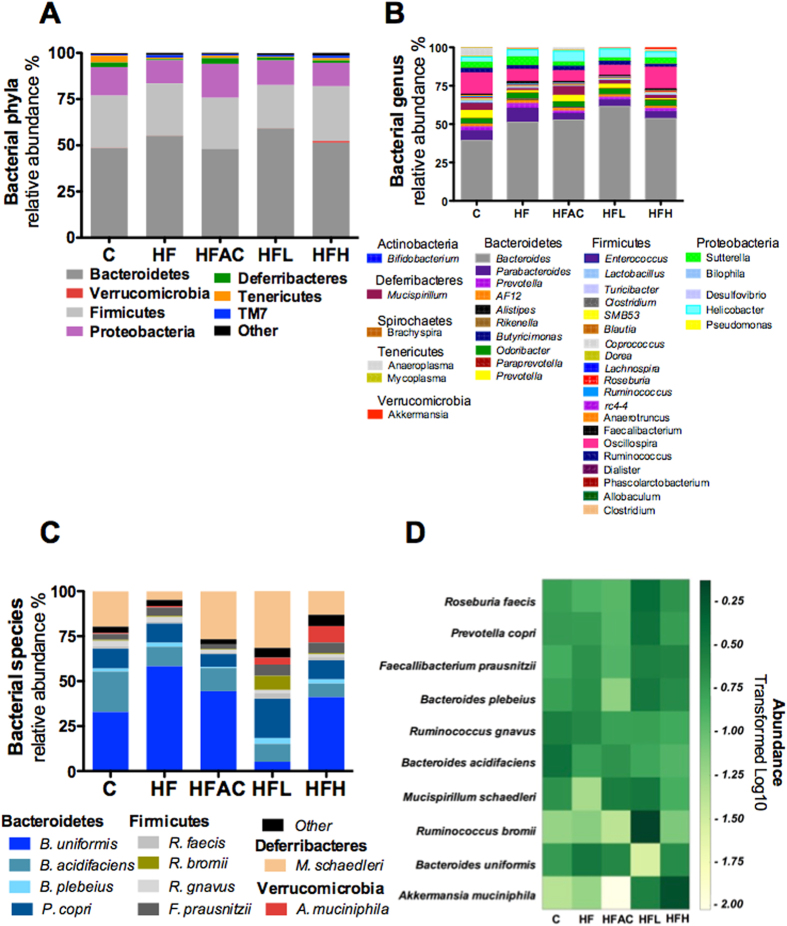
Aguamiel saponins modified the intestinal microbiota and promoted *Akkermansia muciniphila* enrichment. Relative abundances of the gut microbiota (**A**) at the bacterial phyla level, (**B**) genus level and (**C**) species level. (**D**) Heat map showing the relative abundances of bacterial species that covered 93% of all reads assigned. C: control; HF: High-fat; HFAC: High-fat with aguamiel concentrate; HFL: High-fat with low saponin dose; HFH: High-fat with high saponin dose.

The phylum level analysis revealed that Firmicutes and Bacteroidetes abundances were changed in all groups, although the changes were relatively moderate (Fig. 6A). The group fed HFL had the highest increase in Bacteroidetes and lowest increase in Firmicutes compared to the rest of the groups (*P* < 0.05). Interestingly, there was an approximately 6.1-fold increase in Verrucomicrobia in the group fed HFH (*P* < 0.01). To further characterize differences among groups, a sub-phylum analysis was performed by focusing on the families, genus and species. This analysis revealed differences at the family level (Supplemental Fig. S5) particularly in the Bacteroidacea where the HFL and HFH presented abundances of 46.8 and 38.9%, respectively, compared to the C group (27.6%, *P* < 0.001) and the HF group (34.6%, *P* < 0.05). Additionally, the consumption of a high dose of the saponin extract reduced the abundance of the family Helicobacteraceae from 7.2% in the C group to 4.8% (*P* < 0.001). The family Ruminococcaceae was reduced by the consumption of a HF diet (11%, *P* < 0.001) but its abundance was reestablished by the addition of a high concentration of the saponin extract to the HF diet (15.2%, *P* < 0.001) to a similar extent than the C group (15.1%).

Changes at the genus level (Fig. 6B) were similar to those observed at the family level, with the abundance of the genus Bacteroides increased with the HFL (61.5%, *P* < 0.001) or HFH (53.6%, *P* < 0.001) compared to the C group (39.3%). Interestingly, mice fed the C (5.3%, *P* < 0.001), HFAC (4.2%, *P* < 0.001) or HLH (2.9%, *P* < 0.001) diets had higher abundances of the Prevotella genus than mice fed the HF diet (1.5%). A similar pattern of abundance was observed for the Mucispirillum genus. In contrast, the Oscillospira genus belonging to the Ruminococcaceae family was reduced by the consumption of the HF diet (7.8%, *P* < 0.001), but the addition of a high dose of the saponin extract re-established its abundance (14.1%) to a level similar to the C group (13.9%).

At the species level (Fig. 6C,D), we observed that 10 species contributed >93% of the total sequences. These species were *Bacteroides uniformis*, *Bacteroides acidifaciens*, *Prevotella copri*, *Bacteroides plebeius*, *Mucispirillum schaedleri*, *Faecalibacterium prausnitzii*, *Ruminococcus gnavus*, *Roseburia faecis*, *Ruminococcus bromii* and *Akkermansia muciniphila*. Interestingly, the abundance of *Bacteroides acidifaciens* was reduced in the HF mice compared to the C mice, and the addition of the extract reduced its abundance in a dose-dependent manner (Fig. 6D). We also observed that *Akkermancia muciniphila* was almost negligible in mice from the C and HF groups but the addition of the extract increased its abundance in a dose-dependent manner. With the HFL and HFH, *A. muciniphila* was increased by 5.9-fold and 15.3-fold, respectively, with respect to the C group (*P* < 0.001). The increase in *Akkermansia muciniphila* in the HFL and HFH groups was verified by qPCR (Supplemental Fig. S6).

**Figure 7:**
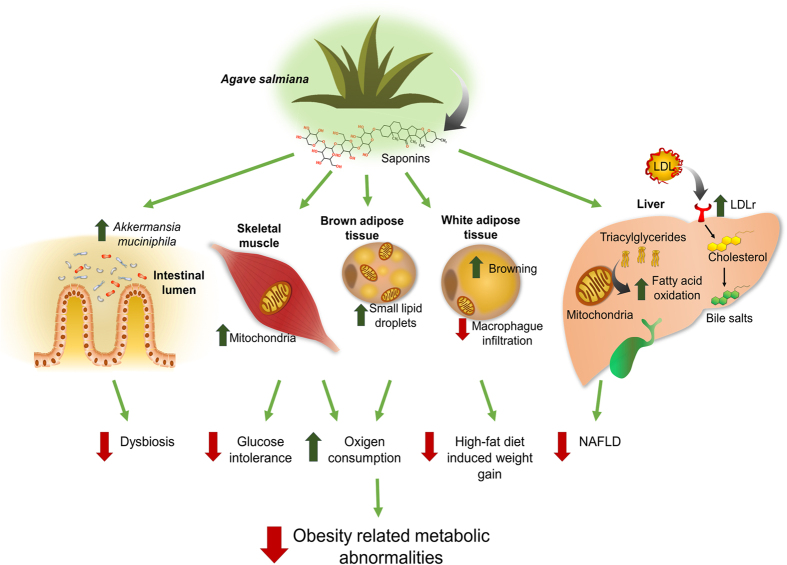
Proposed mechanism of action of aguamiel concentrate and its extracted saponins in diet-induced obesity. AC or its saponin extract exerted beneficial actions in metabolic tissues in mice fed a high-fat diet, thereby improving glucose tolerance and reducing adipose tissue hypertrophy and hepatic steatosis. These effects were mediated by increased WAT browning and mitochondrial activity in skeletal muscle and enhancing the abundance of *Akkermansia muciniphila* in the intestinal lumen. Altogether, these changes resulted in enhanced energy expenditure and preventing many metabolic complications associated with obesity.

In this study, we demonstrated that consumption of AC or its saponin extract improved glucose tolerance and hepatic lipid metabolism, reduced hepatic steatosis and adipose tissue hypertrophy, and stimulated WAT browning and increased the mitochondrial activity in skeletal muscle in mice fed a high fat diet. Altogether, these changes resulted in enhanced energy expenditure. The AC saponin extract prevented the HF diet-induced microbiota dysbiosis by enhancing the abundance of *Akkermansia muciniphila* in the intestinal lumen. Recent evidence has consistently demonstrated that the presence of these bacteria is inversely associated with insulin resistance, altered adipose tissue metabolism, the onset of inflammation, and obesity development during diet-induced obesity in mice^34^,^35^. Dietary polyphenols have recently been reported to promote *A. muciniphila* abundance accompanied by protection against obesity-related pathologies^34^. In this study, we demonstrated for the first time that steroidal saponins extracted from *Agave salmiana* also promoted *A. muciniphila* abundance. Thus, AC or its saponin extract decreased obesity-related metabolic abnormalities (Fig. 7).

In summary, the beneficial effects of the consumption of the *Agave salmiana* aguamiel concentrate and its extracted saponins can be used as part of the dietary strategy to ameliorate the metabolic abnormalities observed in obese subjects. Further studies are needed to evaluate the changes in energy expenditure, insulin sensitivity and the gut microbiota in humans and to use either AC or its saponin extract as part of the strategy to treat obesity.

## Methods

### Glycemic index

The study was undertaken following the ISO 26642:2010 standard method^36^ at the Department of Physiology of the INCMNSZ. Healthy subjects (5 males and 6 females) 29 ± 8 years old with a BMI of 22.5 ± 2.5 participated in the study. After a 10–12 h fast, volunteers consumed the reference (50 g of glucose) or 108 g of AC to meet the 50 g of available carbohydrates. Glucose was analyzed from finger capillary blood with the biochemical analyzer YSI 2700 (Yellow Springs, OH, USA). Insulin was analyzed from venous blood from 3 subjects using a human insulin-specific RIA kit (Human RIA kit, LINCO Research Inc., St. Charles, MO, USA). The ethics committee of the INCMNSZ approved the study, and all subjects gave written informed consent. All procedures were performed in accordance with the ethical standards of the ethical committee on human experimentation of the INCMNSZ and with the Helsinki Declaration of 1975, as revised in 2000.

### AC sugar profile

AC was diluted 16-fold with distilled water and centrifuged (13,800 × g for 10 min at 4 °C). The supernatant sugar profile was determined in an Agilent 1200 Series HPLC-ELSD system using an Xbridge Amide column. The elution profile is available in the supplementary data. The AC nutritional information is available in Supplemental Table S1.

### Saponin extraction

To obtain the saponin crude extract, n-butanol-distilled water (1:1) was mixed with 10% aguamiel concentrate (w/v) and the organic phase was dried under a vacuum. The AC saponin extract preparation is presented in the supplemental methods. The saponin extract was further characterized to determine the total saponin content with an Agilent Technologies 1100 Model HPLC-MS-TOF and Agilent Technologies 1200 Series HPLC-ELSD following the previously reported method^11^.

### Diet-induced obesity model

Male C57BL/6 mice were obtained from the Experimental Research Department at the INCMNSZ, México City, Mexico. Thirty-five mice (5 weeks old and 17–22 g body weight) were assigned to five treatment groups (n = 7). The animals were housed under constant 12 h light/dark cycles at 22 ± 2 °C. The animals had free access to water and food (except where noted). The experimental diets were prepared according to the AIN-93 diet for rodents^37^ and are presented in Supplemental Table S2. The control (C) diet was based on AIN-93, the high-fat (HF) diet was based on AIN-93 with 45% of the kcal from fat, the HF diet was supplemented with 5% AC (HFAC), the HF diet was supplemented with a low dose of AC by adding 2.8 g saponin extract/kg diet (HFL), and the HF diet was supplemented with a high saponin dose (HFH) by adding 28 g saponin extract/kg diet. Food consumption was recorded 5 times per week during the first 10 weeks, and weight gain was recorded twice per week until the end of the study. At the 12^th^ week of the study, the mice were sacrificed with inhaled sevofluorane after 8 h of food deprivation. Blood was collected via the portal vein in heparinized tubes and centrifuged (1000 × g for 10 min at 4 °C), and the plasma supernatant was collected and stored at −80 °C prior to the analysis. The liver and the subcutaneous, retroperitoneal and epididymal fat pads were rapidly excised and weighed. In a 2^nd^ animal study, 5 week old C57BL6 male mice (n = 7) were fed the HF diet for 16 weeks and then switched to the HFH diet for another 8 weeks. Their weight was recorded twice per week. The animal protocols were approved by the Institutional Animal Care and Research Advisory Committee of the INCMNSZ in Mexico City, Mexico and performed in accordance with the Mexican Legislation Regarding the Use and Care of Laboratory Animals.

### Oral glucose tolerance test (OGTT)

In the 1^st^ experiment an OGTT was performed during the 10^th^ week of treatment, and in the 2^nd^ experiment the OGTT was performed at weeks 16 and 24. The glucose load (2 g/kg) was determined using a gavage after 6 h of fasting^35^. Blood glucose was determined using a Freestyle Optium blood glucose monitoring system (Abbot Laboratories, Abbot Park, IL, USA) with blood samples collected from the tail vein at 0, 15, 30, 45, 60, 90, and 120 min after glucose administration^35^. The AUC was calculated using the trapezoid rule.

### Plasma biochemical parameters

The plasma triacylglyceride, total cholesterol, LDL cholesterol, HDL cholesterol, and alanine aminotransaminase (ALT) levels were analyzed using a COBAS C111 analyzer (Roche, Basel, Switzerland). The plasma insulin and leptin levels were measured using commercial ELISA kits (ALPCO, Salem, NH, USA) following the manufacturer’s protocols. Insulin resistance was estimated indirectly through HOMA-IR and calculated as follows: (fasting glucose (mmol/L)) × (fasting insulin (μU/mL))/22.5^38^.

### Liver lipid and malondialdehyde (MDA) analysis

Total lipids were extracted twice from homogenized liver tissue (100 mg) with 3 mL of chloroform–methanol (2:1) and the lower phase was dried under nitrogen^39^. The hepatic lipids were dissolved in isopropanol-Triton X-100 (10%) and assayed to assess the triacylgliceride concentration using enzymatic kits according to the manufacturer’s protocol (DiaSys Diagnostic Systems GmbH, Holzheim, Germany). Liver MDA was measured spectrophotometrically at 586 nm as previously described^40^ and expressed in mmoles of MDA per mg of protein.

### Indirect calorimetry measurement

Animals were individually housed for 48 h in plexiglass cages with an open flow system connected to an Oxymax Laboratory Animal Monitoring System (CLAMS, Columbus Instruments, Columbus, OH, USA). The animals were acclimatized for 24 h, fasted for 6 h during the light period and fed during the dark period. Throughout the test, the volumes of O_2_ consumption (VO_2_, mL/kg/h) and CO_2_ production (VCO_2_, mL/kg/h) were measured sequentially for 90 s. The respiratory exchange ratio (RER) was calculated as the average ratio of CO_2_ produced to O_2_ inhaled (VCO_2_/VO_2_).

### Histological analysis

Samples of liver and adipose tissues (subcutaneous, epididymal, retroperitoneal and brown) were immediately fixed in 10% formaldehyde, embedded in paraffin and cut into 4 μm (liver and BAT) or 6 μm (WAT) slices. For each sample, two sections were stained with hematoxylin and eosin (H&E). The samples were observed under a microscope (Leica DM750 Wetzlar, Germany), photographed with a digital camera (Leica DMC2900), and processed with the imaging software Leica LAS Core V4.5. Analysis of adipocyte area of at least 100 adipocytes per section was done using the Adiposoft software for Image J (ImageJ, NIH)^41^. To visualize the hepatic neutral lipids, frozen liver tissues were sectioned with a cryostat (8 μm) and stained with Oil Red O (ORO) at 0.5% in propylene glycol (Sigma-Aldrich, St. Louis, MO, USA). For the quantitative analysis of the ORO staining, images were converted to an 8-bit grayscale in ImageJ as described^42^ and the integrated density was measured, which is the product of area and mean gray value.

### Immunohistochemistry

F4/80, UCP1 and TNF-α expressing cells were determined in 4 *μ*m thick sections of available formalin-fixed paraffin embedded tissue. Endogenous peroxidase and binding of nonspecific proteins were blocked with 3% H_2_O_2_ and serum-free blocking solution (Enzo Life Sciences, Inc, Farmingdale, NY, USA), respectively. Tissues were incubated with biotinylated rat monoclonal antibodies (eBioscience, San Diego, CA, USA) diluted 1:500 for 18 h at 4 °C. Binding was identified with horseradish peroxidase-(HRP-)streptavidin (ABC Staining System; Santa Cruz Biotechnology). Slides were incubated with substrate 3,3′-diaminobenzidine (DAB) (SIGMA-Aldrich) for 10 min. The sections were counterstained with hematoxylin, dehydrated and mounted in resin. Negative control staining was performed with normal donkey serum diluted 1:100, instead of primary antibody. The reactive blank was incubated with phosphate buffer saline-egg albumin (SIGMA-Aldrich) instead of the primary antibody. Both controls excluded nonspecific staining or endogenous enzymatic activities.

### Muscle succinate dehydrogenase (SDH) activity

The gastrocnemius and soleus muscles were dissected together and immediately frozen in isopentane cooled by liquid nitrogen. Frozen sections (12-μm) were stained and incubated at 37 °C for 60 min. The staining solution was prepared with sodium succinate (270 mg) and nitroblue tetrazolium (10 mg) dissolved in 10 mL of 50 mM PBS (pH 7.5)^43^,^44^. Afterwards, the slides were washed with deionized water and sequentially dehydrated (2 min) with 30, 60 and 90% acetone. Then, the slides were rehydrated (2 min) with 60% and 30% acetone and deionized water^40^,^41^. Digital photographs were taken from each section at 20X magnification as previously described, and fibers were quantified with Image J. SDH activity was quantified by converting the image to 8-bit grayscale and measuring the grey intensity of each fiber.

### Fecal microbiota analysis

Feces were collected for the gut microbial analysis. Total DNA was isolated from frozen fecal samples using the QIAamp DNA Mini Kit (Qiagen, Valencia, CA, USA) according to the manufacturer’s instructions. The DNA eluate was stored at −80 °C prior to sequencing. The microbial community composition analysis was performed using the Illumina MiSeq System to sequence the variable V3 and V4 regions of the 16S rRNA gene according to the protocol suggested by Illumina (16S Metagenomic Sequencing Library Preparation). The overlapping paired-end reads were merged using fastq-join and processed with QIIME V.1.9. (Caporaso JG, 2010). Only Illumina reads with an average score greater than 20 were retained for further analysis. The reads were checked with Chimera and assigned to operational taxonomic units (OTUs) using usearch V5.2.236 (Edgar RC, 2010) with a 97% similarity threshold. Thus, 98.6%, 97.8%, 97.0%, 82.4%, 58.3% and 11.0% of the reads were assigned to the phylum, class, order, family, genus and species levels, respectively. Species richness (Observed, Chao1) and alpha diversity measurements (Shannon) were calculated, and we estimated the within-sample diversity at a rarefaction depth of 5945 reads per sample. Weighted and unweighted UniFrac distances were used to perform the principal coordinate analysis (PCoA). Differences in the relative abundance at the phyla, family, genus and species level were compared using a student T-test for independent samples.

### Quantitative real-time PCR assay

Total RNA was extracted from the liver and subcutaneous adipose tissue with the TRIzol reagent according to the manufacturer’s protocol (Invitrogen, Carlsbad, CA, USA). Reverse transcription for cDNA synthesis and quantitative real-time PCR analysis were performed per mouse as previously described^45^. The tested primer sequences are presented in Supplemental Table S3. Gene expression was normalized to the expression of the housekeeping gene 36B4. The relative expression levels were calculated using the 2−ΔΔCt method^46^. The 16S ribosomal DNA primers sequences used to quantify *Akkermansia muciniphila* are shown in Supplemental Table S4.

### Western blotting assay

Proteins were extracted from homogenized gastrocnemius skeletal muscle using ice-cold RIPA buffer with a Complete Mini protease inhibitor (Roche Diagnostics) and quantified with the Lowry method^47^. Proteins (20 μg) were separated using an SDS-polyacrylamide gel (8%) and transferred to a PVDF membrane. The membranes were blocked for 1 h with 5% non-fat dry milk and incubated overnight at 4 °C in blocking solution with the primary antibody [AMPK1/2 (1:1000), p-AMPK (Thr-172) (1:500), and PGC-1α (1:500) (Santa Cruz Biotechnologies, Santa Cruz, CA)]. Then, the membranes were incubated with a horseradish peroxidase-conjugated secondary antibody (1:3500) for 1.5 h. Visualization was performed using a chemiluminescent detection reagent (Millipore, MA, USA) followed by membrane exposure to film. Β-Actin was used as the loading control. For quantification, densitometric analyses of the immunoblot bands were performed with the ImageJ software.

### Statistical analysis

The experiments were performed per mouse per treatment (n = 7). All analyses were performed in triplicate, and the results were expressed as the mean ± SEM unless otherwise specified. The statistical analyses were conducted with one-way ANOVA, and differences among means were compared with the Bonferroni or Tukey *post hoc* test using a level of significance of *P* < 0.05. The software used was GraphPad Prism version 5.0b.

## Additional Information

**How to cite this article**: Leal-Díaz, A. M. *et al.* Aguamiel concentrate from *Agave salmiana* and its extracted saponins attenuated obesity and hepatic steatosis and increased *Akkermansia muciniphila* in C57BL6 mice. *Sci. Rep.* **6**, 34242; doi: 10.1038/srep34242 (2016).

## Supplementary Material

Supplementary Information

## Figures and Tables

**Table 1:** The effect of aguamiel concentrate or its extracted saponins consumption for 12 weeks on the biochemical parameters in mice.

Parameter	C	HF	HFAC	HFL	HFH
Total cholesterol (mg/dL)	120.9 ± 30.6	146.4 ± 41.6	122.4 ± 29.4	118.4 ± 33.3	118.0 ± 9.5
LDL-cholesterol (mg/dL)	22.4 ± 10.6*	37.5 ± 18.8	23.6 ± 8.9*	21.2 ± 8.6*	20.8 ± 2.4**
HDL-cholesterol (mg/dL)	119.4 ± 27.2	141.3 ± 32.7	122.8 ± 26.2	119.1 ± 31.2	123.1 ± 7.9
Triacylglycerides (mg/dL)	100.9 ± 16.2	90.0 ± 21.5	90.8 ± 25.4	92.7 ± 22.11	74.8 ± 33.0
Glucose (mg/dL)	255.4 ± 43.7	282.3 ± 38.7	284.0 ± 45.0	279.7 ± 53.6	256.9 ± 32.5
Insulin (ng/dL)	1.29 ± 0.3**	1.44 ± 0.5	1.11 ± 0.1***	1.17 ± 0.2***	1.00 ± 0.0***
HOMA-IR	20.2 ± 5.6	25.1 ± 13.2	19.14 ± 4.0	19.7 ± 6.0	15.3 ± 2.0*

Data are presented as mean ± STD DEV (n = 7). Statistical analysis was done with one-way ANOVA followed by Dunnett’s *post hoc* test compared to the HF group. Significance *P < 0.05, **P < *0.01*, ***P < 0.001. C: control; HF: High-fat; HFAC: High-fat with aguamiel concentrate; HFL: High-fat with low saponin dose; HFH: High-fat with high saponin dose.
