# Supplementary material for: Aguamiel concentrate from Agave salmiana and its extracted saponins attenuated obesity and hepatic steatosis and increased Akkermansia muciniphila in C57BL6 mice
Source: Sci Rep. 2016 Sep 28;6:34242. doi: 10.1038/srep34242 (PMC5039706; doi:10.1038/srep34242)
Supplement: Supplementary Information [file srep34242-s1.pdf]

Supplementary information

Title: Aguamiel concentrate from Agave salmiana and its extracted saponins attenuated obesity and hepatic steatosis and increased *Akkermansia muciniphila* in C57BL6 mice

Ana María Leal-Díaz[1], Lilia G. Noriega[2], Ivan Torre-Villalvazo[2], Nimbe Torres[2], Gabriela Alemán-Escondrillas [2], Patricia Lopez-Romero[2], Monica Sanchez-Tapia [2], Miriam Aguilar-López[2], Janette Furuzawa-Carballeda[3], Laura A. Velázquez-Villegas[2], Azalia Avila-Nava[2], Guillermo Ordáz[2], Janet A. Gutierrez-Uribe[1], Sergio O. Serna-Saldivar[1], Armando R. Tovar*[2]

[1] Centro de Biotecnología FEMSA, Tecnológico de Monterrey, 64849, Monterrey, NL, Mexico

[2] Departamento de Fisiología de la Nutrición, Instituto Nacional de Ciencias Médicas y Nutrición Salvador Zubirán, 14000, México D.F., Mexico

[3]Departamento de Inmunología y Reumatología, Instituto Nacional de Ciencias Médicas y Nutrición Salvador Zubirán, 14000, México D.F., Mexico

List of supplementary information

Page S3 - **Table S1**. Aguamiel concentrate nutritional information.

Page S4- **Figure S1.** Effect of aguamiel concentrate and its extracted saponins on the fecal crude fat.

Page S5- **Figure S2**. Aguamiel saponin extract decreased obesity and hyperglycemia in diet-induced obese C57BL6 mice.

Page S6- **Figure S3**. Positive correlation of the liver triacylglycerides (TAG) and the enzyme alanine aminotransaminase (ALT).

Page S7- **Figure S4.** Effect of aguamiel concentrate (AC) and its saponin extract on the retroperitoneal adipose tissue of mice fed a high-fat diet.

Page S8- **Figure S5.** Effect of the diet on the relative abundances of the gut microbiota at the family level.

Page S9- **Figure S6.** Effect of the diet on the relative abundances Akkermansia muciniphila measured by qRTPCR.

Page S10- **Method**. Butanolic crude saponin extraction.

Page S12- **Table S2.** Experimental diet composition offered to C57BL/6 mice during 12 weeks.

Page S14- **Table S3.** Primer sequence used to analyze the liver gene expression.

Page S15- **Table S4.** 16S DNA primer sequence used to analyzed fecal microbiota.

Table S1. Aguamiel concentrate nutritional information

Analysis	Content (g/100g AC)	Method
**Minerals (g)**	10.61	NMX-F-607-NORMEX-2002
**Dietary fiber**	ND	NOM-086-SSA1-1994.AP.C7
**Fat**	ND	NOM-086-SSA1-1994- AP.C1.1.3
**Moisture**	40.84	NOM-116-SSA1-1994
**Protein**	2.25	NMX-F-608-NORMEX-2011
**Available carbohydrates**	46.28	Calculated by difference

Figure S1. Effect of aguamiel concentrate and its extracted saponins on the fecal crude fat. Fecal crude fat was quantified in a Goldfish equipment (Labconco Corporation, MO.) according to the AOAC 920.39 method.[1] Briefly, a pool of mice feces per treatment were dried at 60°C during 12 h. Following the samples (0.3g) were extracted with ether during 6 h. Afterwards, the solvent was evaporated and the crude fat weight was calculated based on the original sample weight. Statistical analysis was done with one-way ANOVA followed by Bonferroni *post hoc* test. Treatments with different letter (a>b) are significantly different at $P<0.05$. C: control; HF: High-fat; HFAC: High-fat with aguamiel concentrate; HFL: High-fat with low saponin dose; HFH: High-fat with high saponin dose.

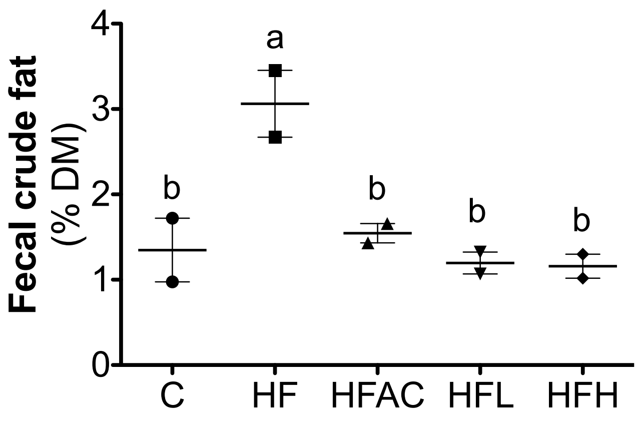

Figure S2. Aguamiel saponin extract decreased obesity and hyperglycemia in diet-induced obese C57BL6 mice. (A) Growth curve of mice fed the HF diet for 16 weeks and then switched to the HFH diet for another 8 weeks, (B) oral glucose tolerance test of mice fed the HF diet (OGTT1) and then switched to the HFH diet for 8 more weeks (OGTT2) and (C) their area under the curve (AUC) values. Data are presented as the mean ± SEM (n=7). The statistical analysis in B was performed by a two-way ANOVA followed by the Bonferroni comparison test (**P*< 0.05, ***P*<0.01). C: control; HF: High-fat; HFAC: High-fat with aguamiel concentrate; HFL: High-fat with low saponin dose; HFH: High-fat with high saponin dose.

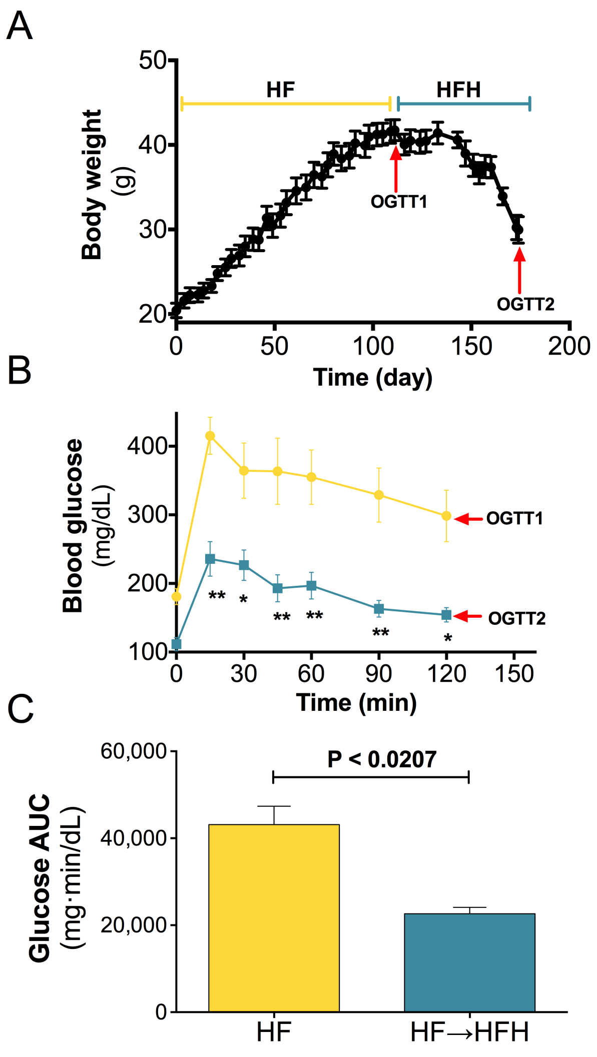

Figure S3. Positive correlation of the liver triacylglycerides (TAG) and the plasma enzyme alanine amino transaminase (ALT).

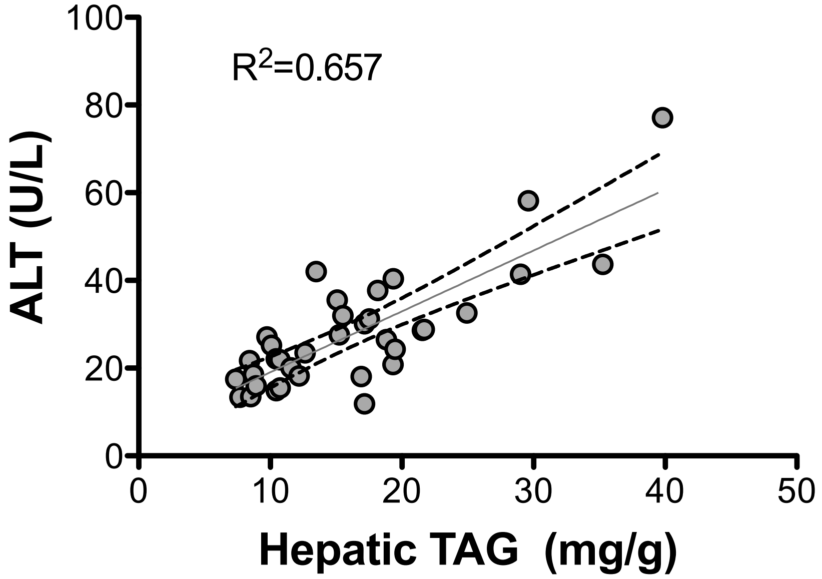

Figure S4. Effect of aguamiel concentrate (AC) and its saponin extract on the retroperitoneal adipose tissue of mice fed a high-fat diet. (A) Hematoxilin and eosin staining of retroperitoneal adipose tissue and (B) adipocyte size distribution. Statistical analysis was done with two-way ANOVA followed by Bonferroni *post hoc*. Mean values with different letters (a>b>c) at the same adipocyte size are significantly different (at least $P<0.05$).

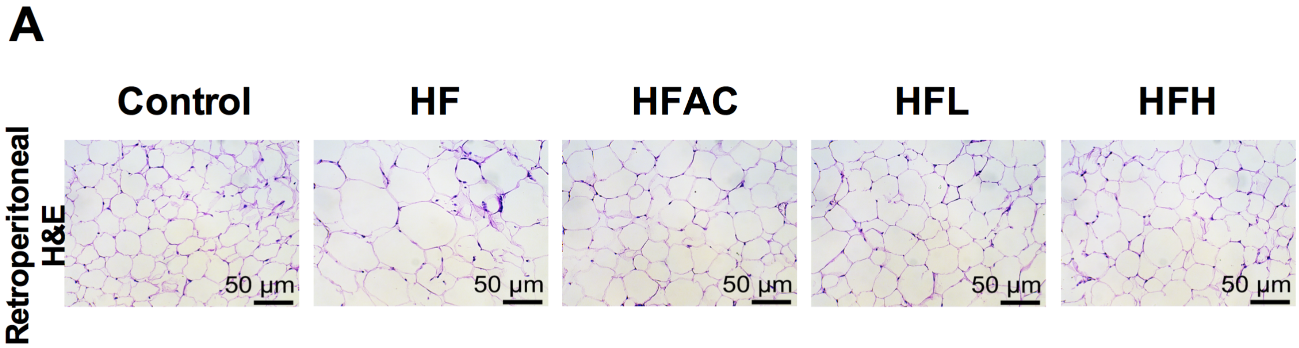

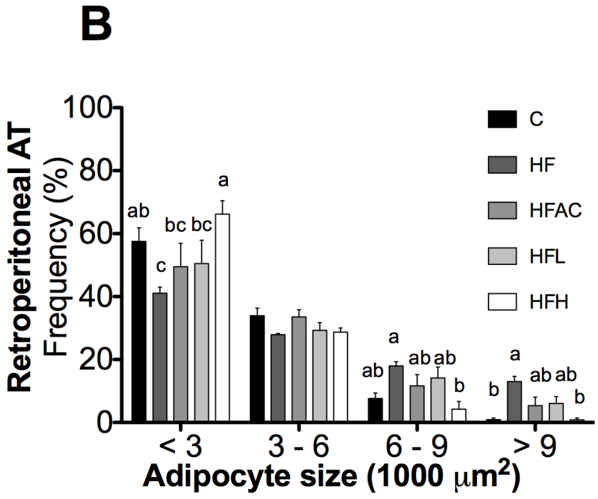

Figure S5. Effect of the diet on the relative abundances of the gut microbiota at the family level. C: control; HF: High-fat; HFAC: High-fat with aguamiel concentrate; HFL: High-fat with low saponin dose; HFH: High-fat with high saponin dose.

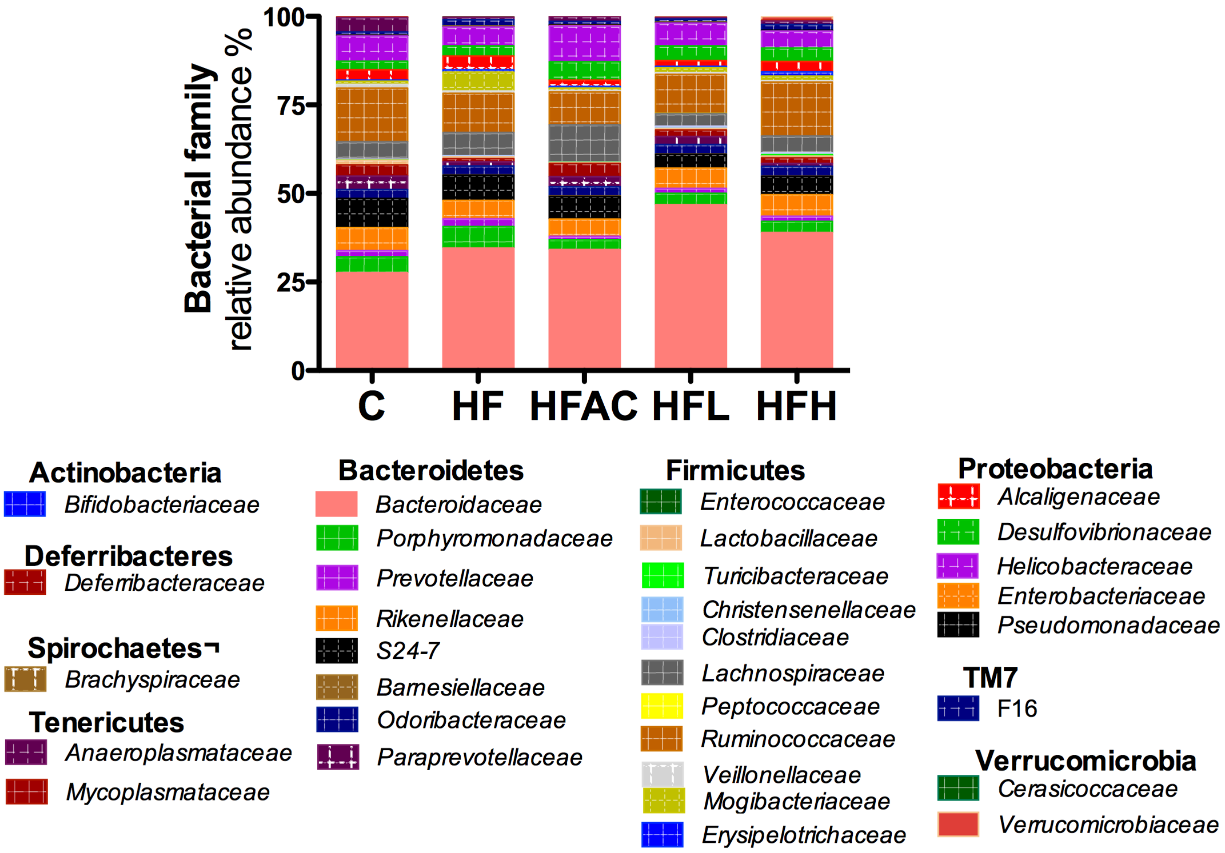

Figure S6. Effect of the diet on the relative abundances *Akkermansia muciniphila* measured by qPCR. C: control; HF: High-fat; HFAC: High-fat with aguamiel concentrate; HFL: High-fat with low saponin dose; HFH: High-fat with high saponin dose.

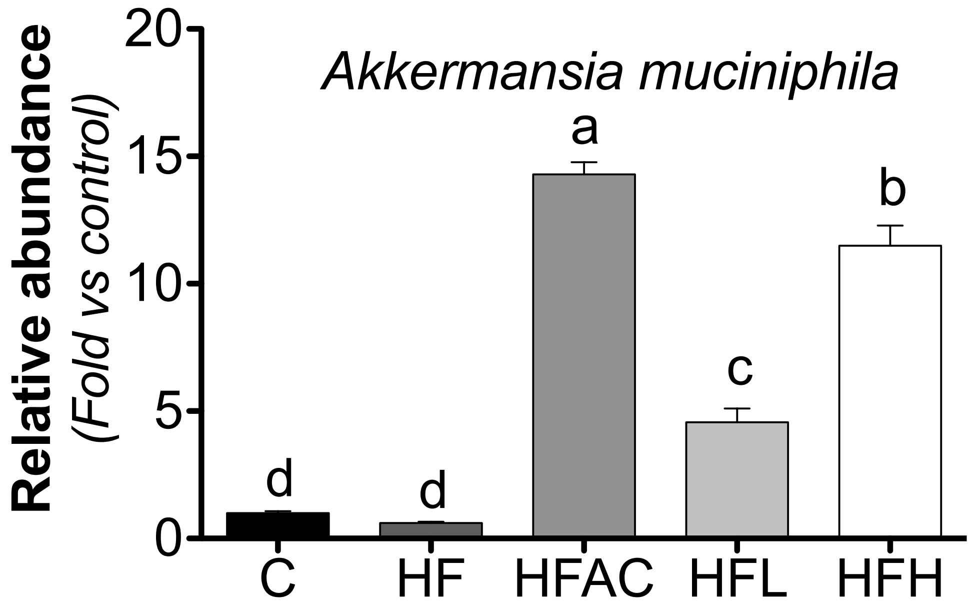

Aguamiel concentrate sugar profiling method.

The aguamiel concentrate (AC) was diluted 16-fold with distilled water. To eliminate any suspended particles, the dilution was centrifuged (Centrifuge 5804 R, Eppendorf, Hamburg, Germany) at 13,800*g* during 10 min at 4 °C. The supernatant was filtered through 0.22 μm nylon membranes (VWR International LLC, Radnor, PA). Sugar concentrations were quantified using liquid chromatography with evaporative light scattering (HPLC-ELSD) detection (Agilent Technologies, 1200 Series, Santa Clara, CA). Sample (10 µL) was separated in a Xbridge Amide column, 3.5 µm, 4.6 x 250 mm, (Waters Corporation, Milford, MA) with mobile phase A: 80% acetonitrile (ACN) (BDH, Poole, UK) and 20 water (BDH, Poole, UK) with 0.2% triethylamine (Sigma-Aldrich, St. Louis, MO) and B: 30/70 ACN/water with 0.2% triethylamine, at a flow rate 1 mL/min, using the following gradient, starting at 10% B: 0→16 min, 70%B; 16.00→16.1 min, 10%B; 16.1→30 min, 10%B. Conditions for ELSD were: gain 2, pressure 2 bar, temperature 50°C, filter: 0.5 s and sampling 100-10 Hz. D-glucose (Sigma-Aldrich, St. Louis, MO), D-fructose (Merck KGaA, Darmstadt) and sucrose (Fluka, St. Louis, MO) were used as standards, in a range from 0.5 to 10 mg/mL for each sugar. Results are reported as % of sugars[2].

Butanolic crude saponin extract.

To obtain the saponin crude extract, *n*-butanol-distilled water (1:1) with 10% of aguamiel concentrate w/v were agitated at 110 rpm during 2 h in 20L capacity agitated tank (Chemglass, Inc., Vineland NJ). The mixture was then rested for 6 h until the two phases were separated. The organic phase was centrifuged (3,000 g, 10 min and 25°C). The upper phase was collected and the lower phase was added to the remaining aqueous phase for a second extraction. In the second extraction, *n*-butanol was added in the same proportion as the remaining aqueous phase and followed the same procedure as the 1st extraction. The centrifuged upper phase was then concentrated in a 20 L rotary evaporator Rotavapor® R-220, (Büchi, Flawil, Switzerland) at 55°C, 70 rpm and vacuum from 120 to 60 bar. The residual dried extract was dissolved in ethanol 70% and concentrated in a 1 L rotary evaporator IKA RV 10 (IKA, Wilmington, NC) at 55°C, 70 rpm and vacuum from 120 to 60 bar. To validate and quantitate the saponin present in the crude extract, the dried extract dissolved in distilled water and filtered through a syringe filter, pore size 0.45 μm made of hydrophilic polypropylene from Pall Life Science (Ann Arbor, MI). Identification was done with HPLC-MS-TOC-ESI+ and quantitation with HPLC ELDS as previously reported by Leal-Díaz (2015)[3]

Table S2. Experimental diet composition offered to C57BL/6 mice during 12 weeks.

Ingredient (g/Kg)	C[a]	HF[b]	HFAC[c]	HFL[d]	HFH[e]
Corn starch	397.5	247.7	247.7	247.7	247.7
Casein (≥ 85% protein)	200	245	241.8	245	245
Maltodextrin	132	71.3	71.3	71.3	71.3
Sucrose	100	100	76.9	100	100
Soybean oil	70	73.5	73.5	73.5	73.5
Cellulose	50	50	50	50	50
Mineral Mix (AIN-93G)	35	35	35	35	35
Vitamin Mix (AIN-93G)	10	10	10	10	10
L-Cystine	3	3	3	3	3
Choline bitartrate	2.5	2.5	2.5	2.5	2.5
Tert-butylhydroquinone (TBHQ)	0.014	0.046	0.046	0.046	0.046
Lard	-	161.5	161.5	161.5	161.5
Saponin rich extract (DM)	-	-	-	2.8	28
Aguamiel concentrate	-	-	50	-	-
**% Energy (Kcal) from:**					
Protein	19	19	19	19	19
Fat	16	45	45	45	45
Carbohydrates	65	36	36	36	36

C, Control diet based on AIN-93

HF, high-fat diet with 45% of the Kcal coming from fat.

HFAC, high-fat high saponin dose with 45% of the Kcal coming from fat and 5% aguamiel concentrate added. Nutrients coming from the AC were subtracted from the formula.

HFL, high-fat low saponin dose with 45% of the Kcal coming from fat and 2.8g of saponin rich extract with 60 mg saponins HE.

HFH, high-fat high saponin dose with 45% of the Kcal coming from fat and 28g of saponin rich extract with 600 mg saponins HE.

Table S3. Primer sequence used to analyse the liver and subcutaneous adipose tissue gene expression.

Gene	Reverse 5’→3’	Forward 3’→5’
**ABCA1**	GGGAAACAGCCCAGTCAGTA	AGCCAGAAGGGAGTGTCAGA
**ABCG5**	GAAGCCAAGCATCTCCTCTG	GATGAGCCAACCACAGGACT
**ABCG8**	ACGTCGAGTAGTGAGGCTCT	CCTCTCAGGTGCCTTGGTTT
**ACC**	GACTGTGCCTGGAACCTCTT	GCCTCTTCCTGACAAACGAG
**AOX**	CCACATATGACCCCAAGACC	AGGCATGTAACCCGTAGCAC
**CYP7A1**	TGGGCTGTGCTCTAAGTTC	CTGTGTCCAAATGCCTTCGC
**CPT-1**	AAGGTGCTGCTCTCCTACCA	TCATCAGTGGCCTCACAGAC
**FAS**	TACAACAGCCTCAGAGCGAC	CAAAGGACCAAGCATTGCCC
**HMGCR**	GGATTGCCATTCCACGAGCT	GGGTATTGCTGGCCTCTTCA
**LDLr**	GAGCCATCTAGGCAATCTCG	ATGAGTCCCCAGAGACATGC
**PPARα**	GCAGCTCGTACAGGTCATCA	CTCTTCATCCCCAAGCGTAG
**SCD-1**	TAGTCGAAGGGGAAGGTGTG	TGCGATACACTCTGGTGCTC
**SERBP1c**	AAGCGGATGTAGTCGATGGC	AGACAAACTGCCCATCCACC
**SERBP2**	GTCACGAGGCTTTGCACTTG	GATGATCACCCCGACGTTCA
**UCP1**	CTTTGCCTCACTCAGGATTGG	ACTGCCACACCTCCAGTCATT
**TBX1**	TGAGGAGACACGCTTCACTG	CTGCAGCGTCTTTGTCTGAG
**Housekeeping 36B4**	AGATTCGGGATATGCTGTTGG	AAAGCCTGGAAGAAGGAGGTC

Table S4. 16S ribosomal DNA primer sequence used in *Akkermansia muciniphila* qPCR analysis.

Gene	Forward 3’→5’	Reverse 5’→3’
Akkermansia muciniphila	CCTTGCGGTTGGCTTCAGAT	CAGCACGTGAAGGTGGGGAC
**Universal primer**	CTCACRRCACGAGCTGAC	AAACTCAAAGAATTGACGG
